# Endoscopic stenting of malignant, benign and iatrogenic colorectal disorders: a primer for radiologists

**DOI:** 10.1186/s13244-019-0763-1

**Published:** 2019-08-28

**Authors:** Massimo Tonolini, Emilia Bareggi, Raffaele Salerno

**Affiliations:** 1 Department of Radiology, “Luigi Sacco” University Hospital, Via G.B. Grassi 74, 20157 Milan, Italy; 2 Digestive Endoscopy, ASST Fatebenefratelli Sacco, Via G.B. Grassi 74, 20157 Milan, Italy

**Keywords:** Colon, Stent, Colorectal carcinoma, Computed tomography (CT), Magnetic resonance imaging (MRI)

## Abstract

In recent years, endoscopic placement of intraluminal stents is increasingly used to manage a widening range of colorectal disorders. Self-expanding metal stents represent an established alternative to surgery for the palliation of unresectable carcinomas and currently allow a “bridge-to-surgery” strategy to relieve large bowel obstruction and optimise the patients’ clinical conditions before elective oncologic resection. Additionally, intraluminal stents represent an appealing option to manage obstructing extracolonic tumours and selected patients with benign conditions such as refractory anastomotic strictures and post-surgical leaks.

This educational paper reviews the technical features and current indications of colorectal stenting and presents the expected and abnormal radiographic, CT and MRI appearances observed during the endoscopic management of malignant, benign and iatrogenic colonic disorders with stents. The aim is to provide radiologists with a thorough familiarity with stent-related issues, which is crucial for appropriate reconstruction of focused CT images, correct interpretation of early post-procedural studies and elucidation of stent-related complications such as misplacement, haemorrhage, perforation, migration and re-obstruction.

## Key points

- Self-expanding metal stents safely allow both palliative and bridge-to-surgery treatment of obstructing colorectal carcinomas
- Stenting of benign colorectal disorders is mostly reserved for selected refractory anastomotic strictures and post-surgical leaks
- With appropriate reconstructions, CT provides a comprehensive high-resolution visualisation of the stent, the treated colonic disease and surrounding structures
- CT is the technique of choice to comprehensively investigate patients with clinical suspicion of stent-related complications
- The commonest stent-related complications include misplacement, haemorrhage, perforation, migration and re-obstruction

## Introduction

During the past two decades, growing experience and technical advancements progressively widened the indications and increased the use of intraluminal stents in the management of colorectal disorders. Specifically, endoscopic decompression of malignant large bowel obstruction (LBO) by means of self-expanding metal stents (SEMS) progressively gained acceptance as an alternative to surgical resection or diversion, particularly in metastatic tumours and in poor surgical candidates. Initially considered a palliative treatment, SEMS are nowadays used as a “bridge” to elective surgical management of colorectal carcinoma (CRC). Furthermore, endoscopists progressively perform stenting of benign colorectal disorders in selected patients. Finally, the use of stents represents an appealing option in the challenging management of iatrogenic complications following colorectal surgery. The current indications for stent placement to manage different colorectal disorders are summarised in Table 1 and discussed in detail in the following paragraphs [1, 2].

**Table 1:** Indications for stent management of colorectal disorders

Indication	
Palliation of colorectal carcinoma (CRC)-related bowel obstruction In non-operative candidates Non-resectable malignant stricture Local postoperative neoplastic recurrence	
Preoperative decompression in obstructing resectable CRC (bridge-to-surgery)	
Relief of large bowel obstruction from extracolonic malignancy, pelvic mass or peritoneal carcinomatosis	
Malignant colorectal fistula, e.g. to the urinary bladder or vagina	
Postoperative anastomotic leakage/fistula	

Aimed at radiologists practicing emergency and oncologic imaging, this pictorial essay reviews the technical features and current indications of colorectal stenting and presents with examples the expected and abnormal radiographic and cross-sectional imaging appearances observed during endoscopic management of malignant, benign and iatrogenic colonic disorders using intraluminal stents [3, 4].

## Colonic stenting: technical basics

**Fig. 1:**
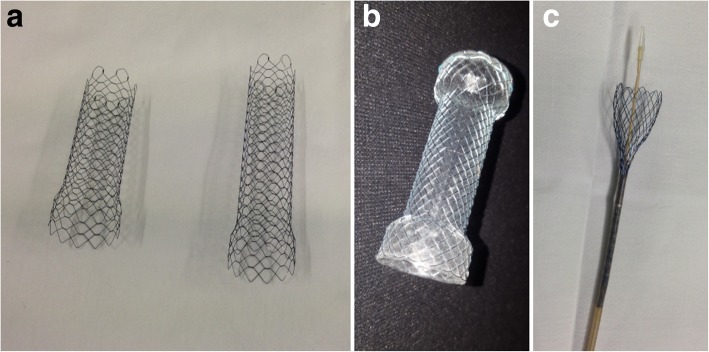
Some common self-expandable metal stents (SEMS) available for use in colorectal disorders. **a** Two opened uncovered WallFlex^TM^ (Boston Scientific, Massachusetts, USA) stents measuring 25 × 60 mm (left side) and 22 × 90 mm (right side), respectively. **b** Fully covered Niti-S stent (Taewoong Medical, South Korea) 22 × 100 mm. **c** Uncovered WallFlex^TM^ stent, closed along its introducer sheath

Stents for use in the large bowel (Fig. 1) are available in variable diameter, length and mural structure, in order to adapt to the colonic lesion or injury to be treated. When opened, SEMS appear as hollow cylindrical devices with a reticular “mesh” pattern, made of stainless steel or metal alloy such as nitinol (Fig. 1a, b). Initially, SEMS are provided tightly compressed along a delivery guidewire (Fig. 1c) that may pass through the operative channel of the endoscope.

Technically, SEMS are categorised as bare, partially (at the centre) or fully covered, depending on the absence or presence of a non-porous chemical “membrane” that prevents tumour growth through the mesh. In the management of obstructing CRC, no significant differences were found between covered and uncovered SEMS in terms of technical and clinical success, perforation and overall complication rates. Compared to covered ones, uncovered SEMS suffer from significantly higher obstruction rates from tumour ingrowth, but are less prone to migration. Conversely, fully covered SEMS allow prolonged stent patency and have a higher likelihood of retrieval due to the limited local tissue reaction [2, 5]. Recently, biodegradable stents made of polydioxanone have been developed, but their use remains controversial [6, 7].

**Fig. 2:**
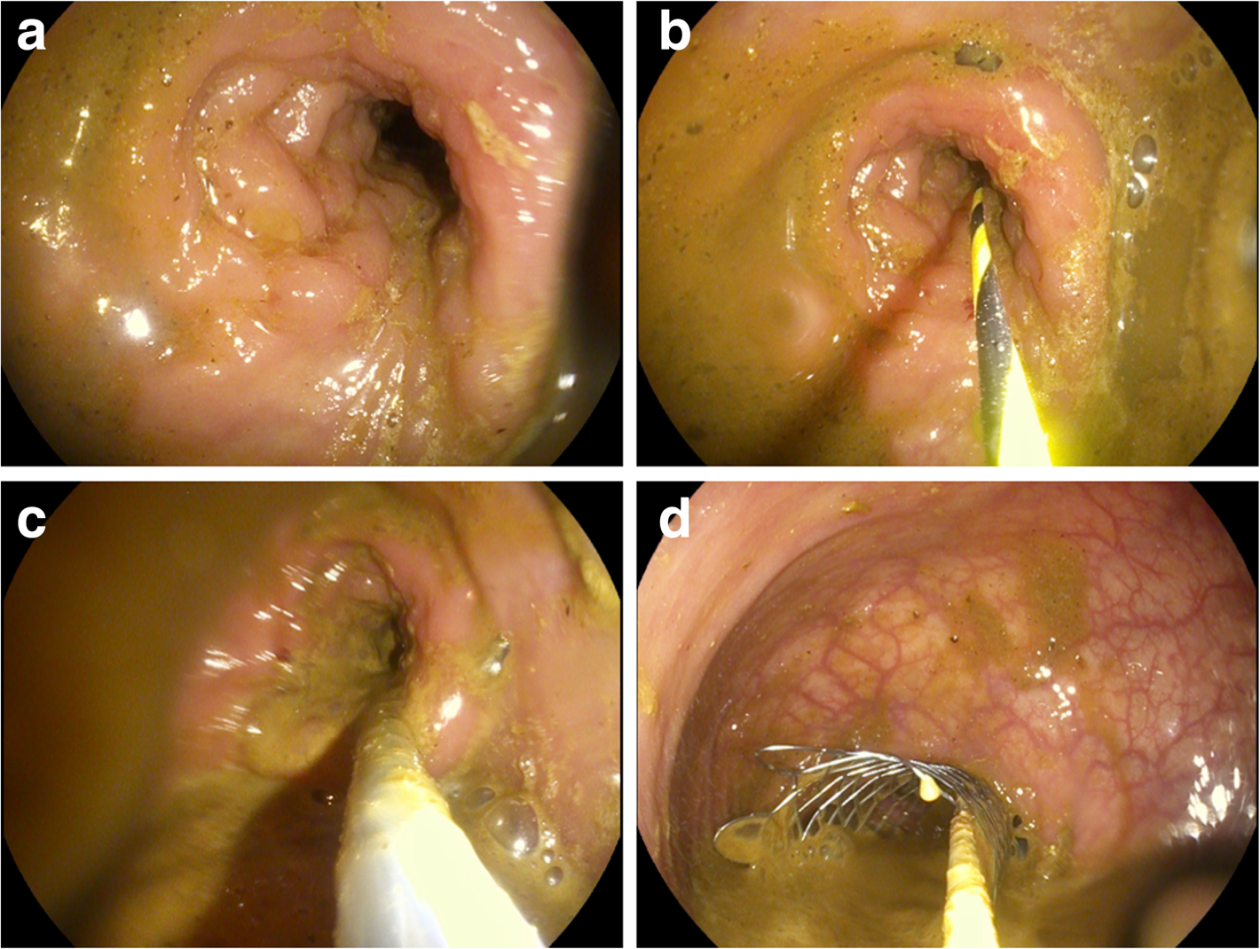
Endoscopic placement of a SEMS through an obstructing tumour of the sigmoid colon. **a** Distal endoscopic view of colorectal carcinoma (CRC) impassable with the endoscope. **b** Under fluoroscopic guidance, the guidewire is passed through the stricture. **c** The SEMS is advanced along the guidewire. **d** After deployment and initial expansion of the SEMS, prompt outflow of faecal content is seen

Stents are positioned in the colonic lumen during endoscopy, generally under fluoroscopic guidance since the metal mesh is radio-opaque (Fig. 2). After the guidewire is passed through the stricture, the SEMS is released using the deployment device at its end and begins expanding. Complete expansion of the SEMS in place usually occurs within 24–48 h and may sometimes require a few days to be completed [2].

## Stents in the management of colorectal carcinoma

### Palliation of malignant large bowel obstruction

**Fig. 3:**
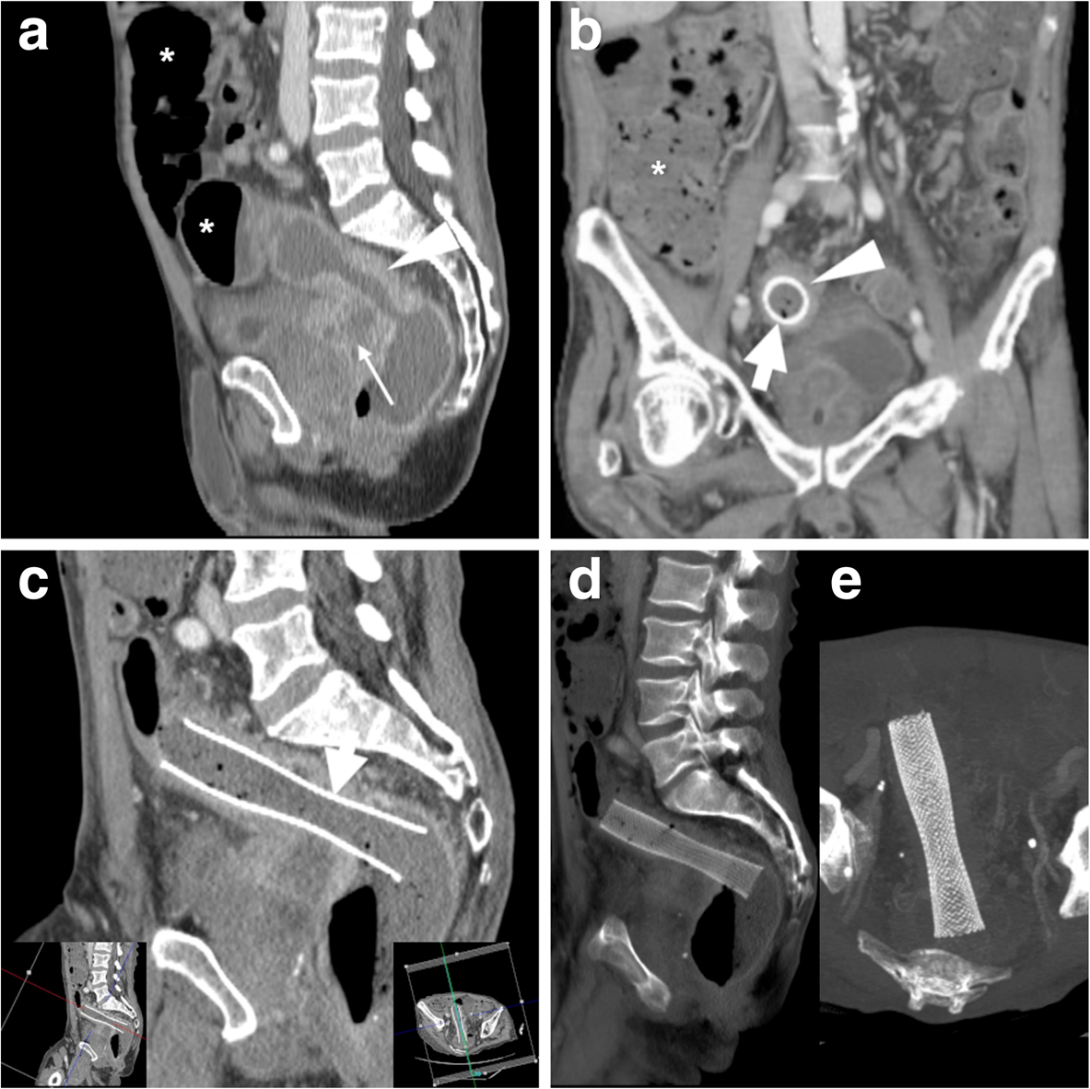
Palliation of unresectable CRC and dedicated CT reconstruction techniques. In an elderly male, sagittal contrast-enhanced CT (**a**) shows a 6-cm-long segment with enhancing, non-stratified increased mural thickness (arrowhead) at the rectosigmoid junction, causing luminal stricture and upstream large bowel obstruction (LBO*) complicated by fistulisation (thin arrow) to the prostate and urinary bladder. After endoscopic stenting, repeated CT (**b**, **c**) including focused oblique images (note obliquity in **c** insets) effectively depicted the correct position of the SEMS (thick arrows) at the site of CRC (arrowheads), filled with faecal fluid with partial persistence of upstream colonic dilatation (*). Additional thick-slab (**d**) and maximum-intensity projection (MIP) reconstructions depict the tubular shape and reticular “mesh” structure of the well-expanded SEMS (**e**)

**Fig. 4:**
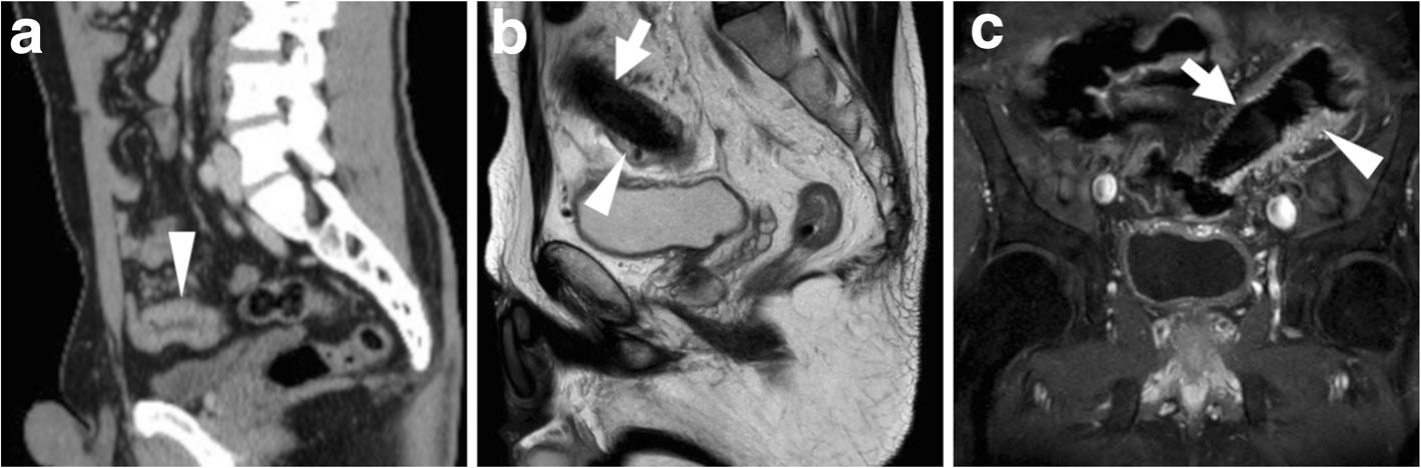
Usual MRI appearance of a SEMS-treated CRC. At diagnosis, sagittal CT image (**a**) shows an endoscopically impassable, ulcerated CRC of the sigmoid colon as segmental mural thickening (arrowhead). After stent placement, sagittal T2- (**b**) and coronal fat-saturated T1-weighted after intravenous gadolinium contrast (**c**) MRI images show well-expanded SEMS (thick arrows) with patent lumen devoid of signal, and mural thickening (arrowheads) with T2-hypersignal (**b**) and positive enhancement (**b**) corresponding to the treated tumour

LBO is not uncommon in emergency departments and is caused by CRC in almost two thirds of cases [8]. Historically, the first indication for colonic stenting has been minimally invasive management of malignant LBO in non-operative candidates and in patients with non-resectable, fistulising or disseminated disease (Figs. 3 and 4). Currently, according to the European Society of Gastrointestinal Endoscopy (ESGE), SEMS placement is the preferred palliative treatment for malignant LBO, with the exception of patients treated with or who will receive antiangiogenic drugs such as bevacizumab, since the presence of the stent determines a threefold increase in the risk (10–20%) of either clinically overt and silent perforations [5].

In CRC patients with very poor lifetime expectancy, SEMS placement improves the quality of life and generally obviates unnecessary surgery and further morbidity. On the other hand, in patients with advanced stage CRC, SEMS placement can avoid a permanent stoma and result in lower morbidity, shorter hospitalisation, earlier start of chemotherapy and similar survival compared to surgery. In the palliative setting, when the expansion is satisfactory after 48 h, 80% and 72% of SEMS remain patent after 6 and 12 months, respectively [9–14].

### Stenting as a “bridge to surgery”

**Fig. 5:**
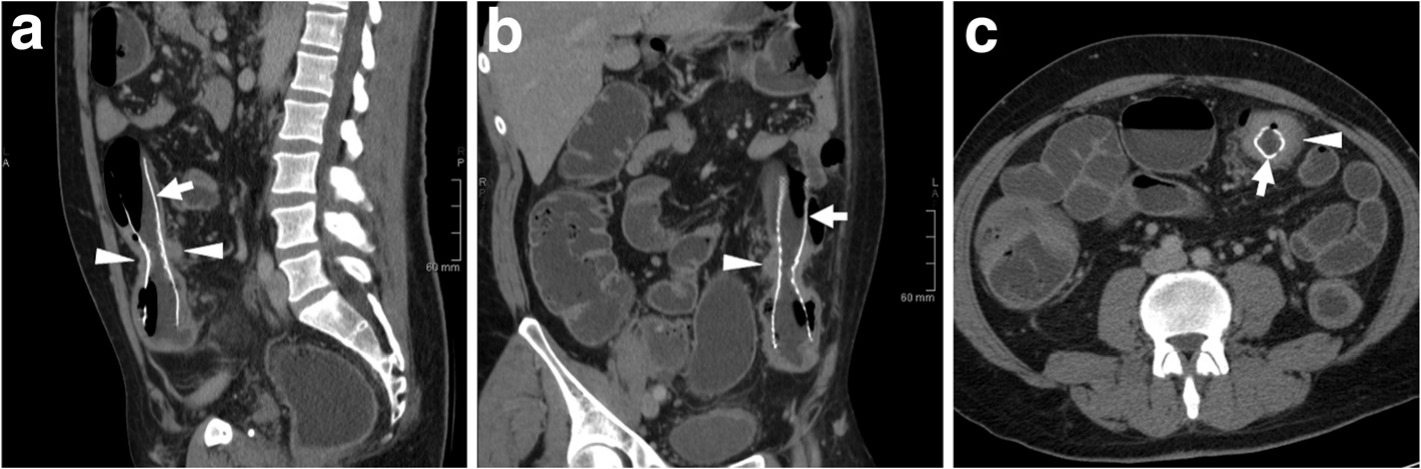
Comprehensive staging of SEMS-treated CRC using water enema CT. Multiplanar contrast-enhanced images (**a**–**c**) show sufficiently expanded SEMS (thick arrows) at a stricturing tumour of the descending colon, seen as circumferential enhancing mural thickening (arrowheads). The upstream large bowel is adequately distended by water and does not show additional suspicious changes

Traditionally, immediate surgical resection of obstructing CRC was burdened with high morbidity (40–60%) and substantial postoperative mortality (8–20%). Furthermore, one-stage surgery without a colostomy is technically difficult in the emergency setting, resulting in a high risk of a temporary or permanent stoma. Nowadays, SEMS have emerged as an effective alternative option to manage malignant LBO that facilitates subsequent elective surgery with primary anastomosis. Early colonic decompression by SEMS placement allows time to optimise the patient’s medical conditions, to take biopsies and stage the CRC (Fig. 5), to obtain preoperative bowel cleansing and sometimes even to administer neoadjuvant chemotherapy. During elective one-stage surgery (usually performed 8 to 10 days after stent placement), the SEMS is surgically removed en bloc with the colon specimen [14].

Indications for this “bridge-to-surgery” approach include patients with potentially curable left-sided CRC with symptoms, clinical and imaging signs of LBO, particularly those with an increased risk of postoperative mortality according to age and American Society of Anesthesiologists (ASA) Physical Status classification system. Exclusion criteria include poor life expectancy, bowel perforation and haemorrhage. Stenting of rectal carcinomas is generally avoided because of the higher risk of complications such as pain, tenesmus, incontinence and stent migration [1, 2, 5, 14].

**Fig. 6:**
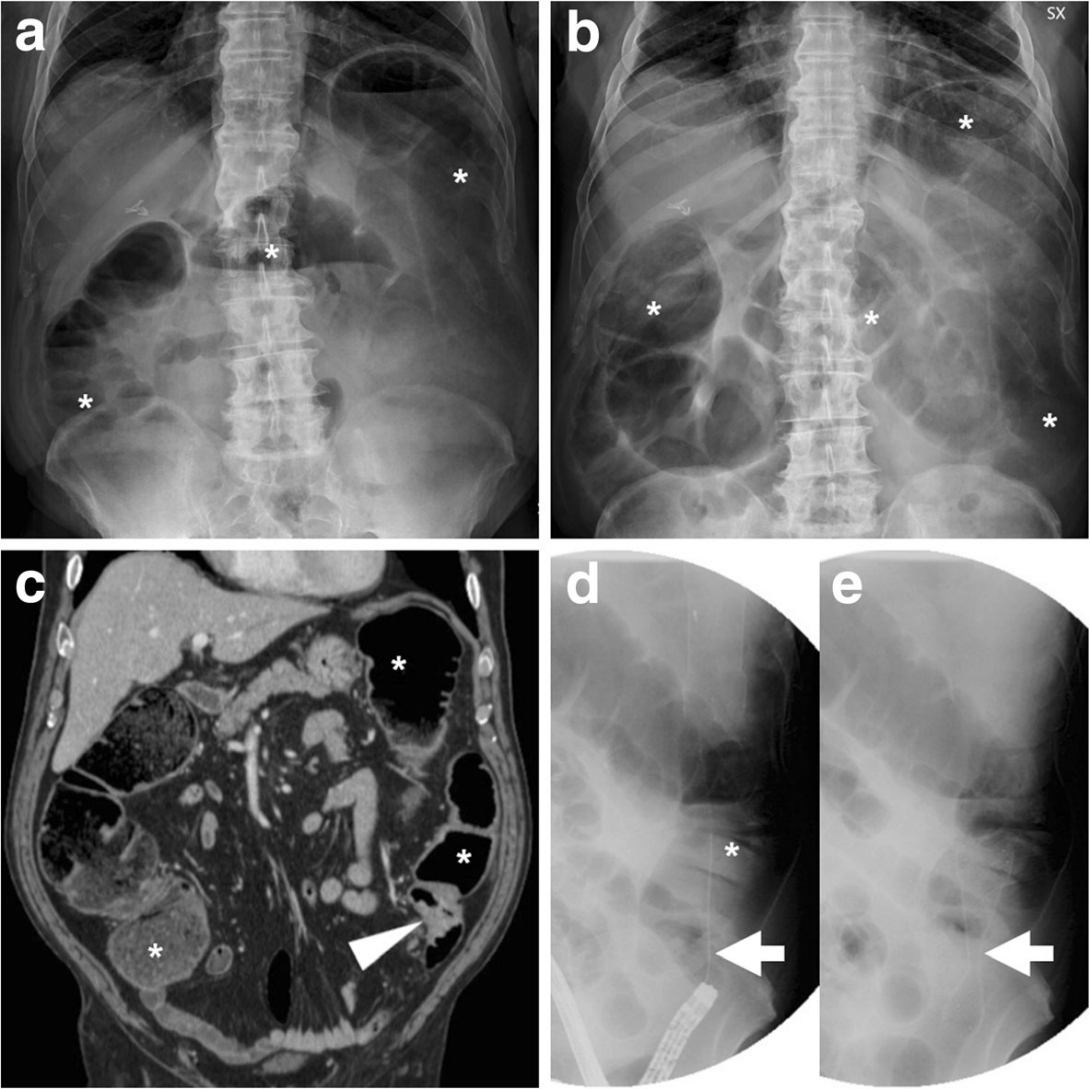
“Bridge-to-surgery” stenting of obstructing CRC. Upright (**a**) and supine (**b**) plain abdominal radiographs show marked dilatation of the large bowel (*) with fluid-faecaloid material consistent with mechanical LBO (note air-fluid levels in **a**). Coronal CT image (**c**) confirm colonic dilatation (*) above a 4-cm-long, solid mural thickening at the distal descending colon (arrowhead). After biopsy, fluoroscopic images documented the endoscopic positioning of a SEMS (thick arrow in **e**) along the guidewire (thick arrow in **d**) through the tumour. The patient then underwent left hemicolectomy with primary anastomosis to remove pT3N1G2 carcinoma

**Fig. 7:**
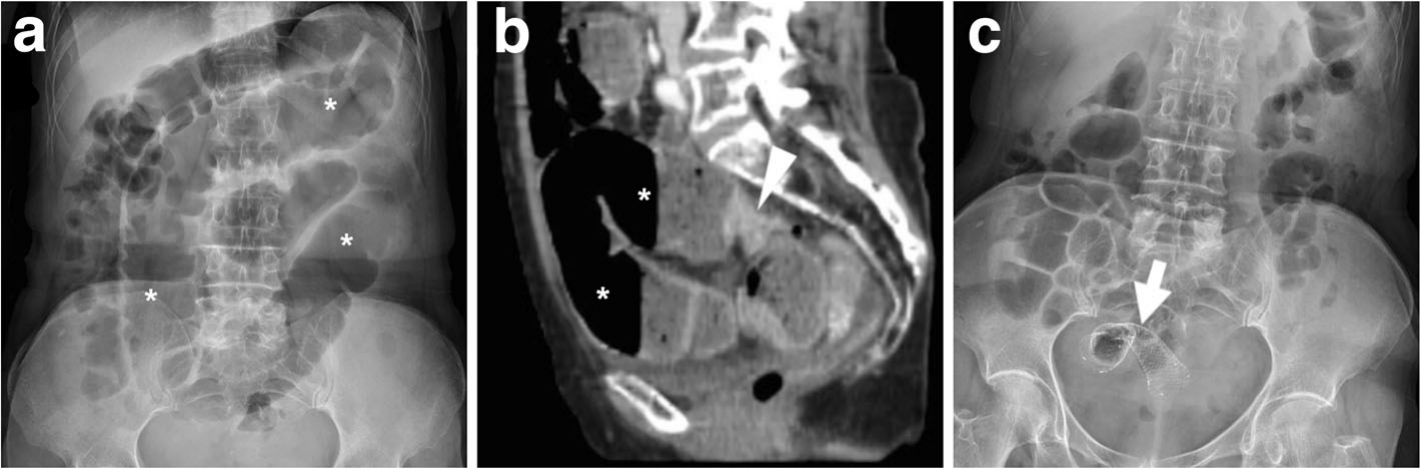
Effective relief of LBO during bridge-to-surgery stenting of CRC. Initial supine (**a**) radiograph shows marked gaseous dilatation of the large bowel (*) consistent with LBO. Sagittal post-contrast CT image (**b**) confirm air-fluid colonic dilatation (*) above a short stricturing sigmoid tumour (arrowheads). Forty-eight hours after endoscopic positioning of a SEMS (thick arrow), a plain radiograph (**c**) shows near-complete resolution of LBO. Three weeks later, the patient successfully underwent sigmoid resection with termino-terminal anastomosis

**Fig. 8:**
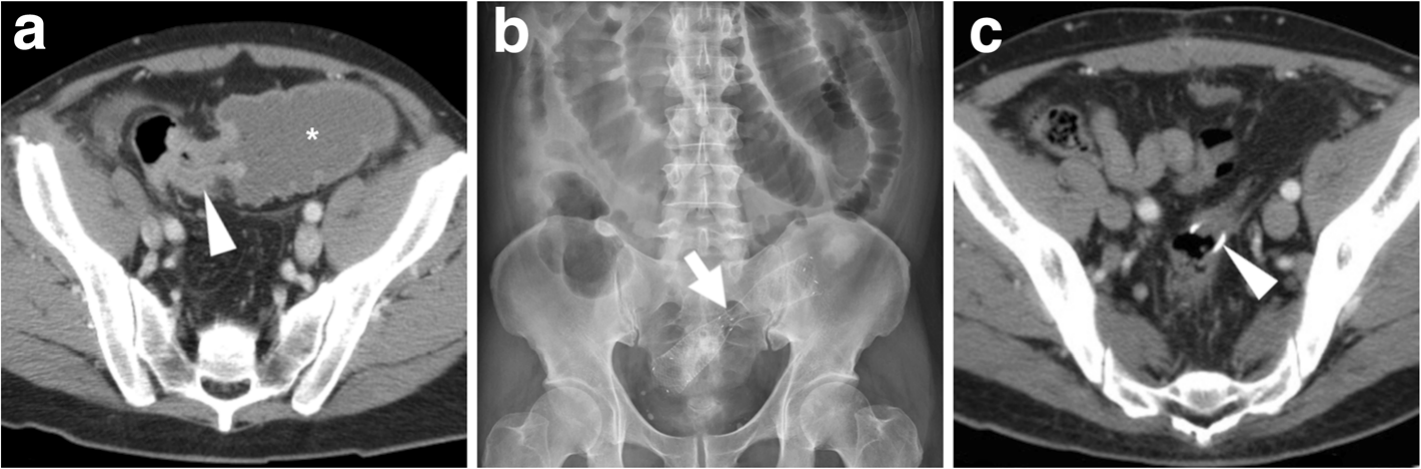
Delayed relief of LBO during bridge-to-surgery stenting of CRC. Initial CT (**a**) shows luminal dilatation (*) above a stricturing tumour of the sigmoid colon (arrowhead, note shouldering). Early radiograph (**b**) shows persistent colonic dilatation despite correct placement of the SEMS (thick arrow) at the site of CRC. The patient experienced slow clinical and radiographic improvement and had neoadjuvant chemotherapy followed by resection of pT3N2 cancer with primary anastomosis. Note suture on early postoperative CT (**c**) performed to exclude anastomotic dehiscence

Bridge-to-surgery management of obstructing CRC (Figs. 6, 7 and 8) has proven to be highly effective and safe, with limited procedure-related complications. Technical success (corresponding to appropriate insertion of the SEMS across the entire length of the stenosis) is achieved in 90% of CRC-related obstructions. Failure most commonly results from the inability to pass the guidewire across a tortuous or severe stricture. Clinical success, defined as effective resolution of LBO with symptomatic relief, is achieved in over 70% of patients within the first days after SEMS placement. Compared to surgery alone, bridge-to-surgery SEMS result in lower operative time, lower overall complication rates (33.9–37.8% versus 51.2–54.8%) including less wound infections, shorter or equal intensive care unit stay, similar early mortality, lower rates of temporary (28.8–33.9% versus 46–51.4%) and permanent (22.2% versus 35.3%) stoma [15–17].

The long-term outcome is similar between patients undergoing emergency surgery and those who receive preoperative SEMS, regarding recurrence rates, overall and disease-free survival [18, 19].

## Stent management of extracolonic malignancies

**Fig. 9:**
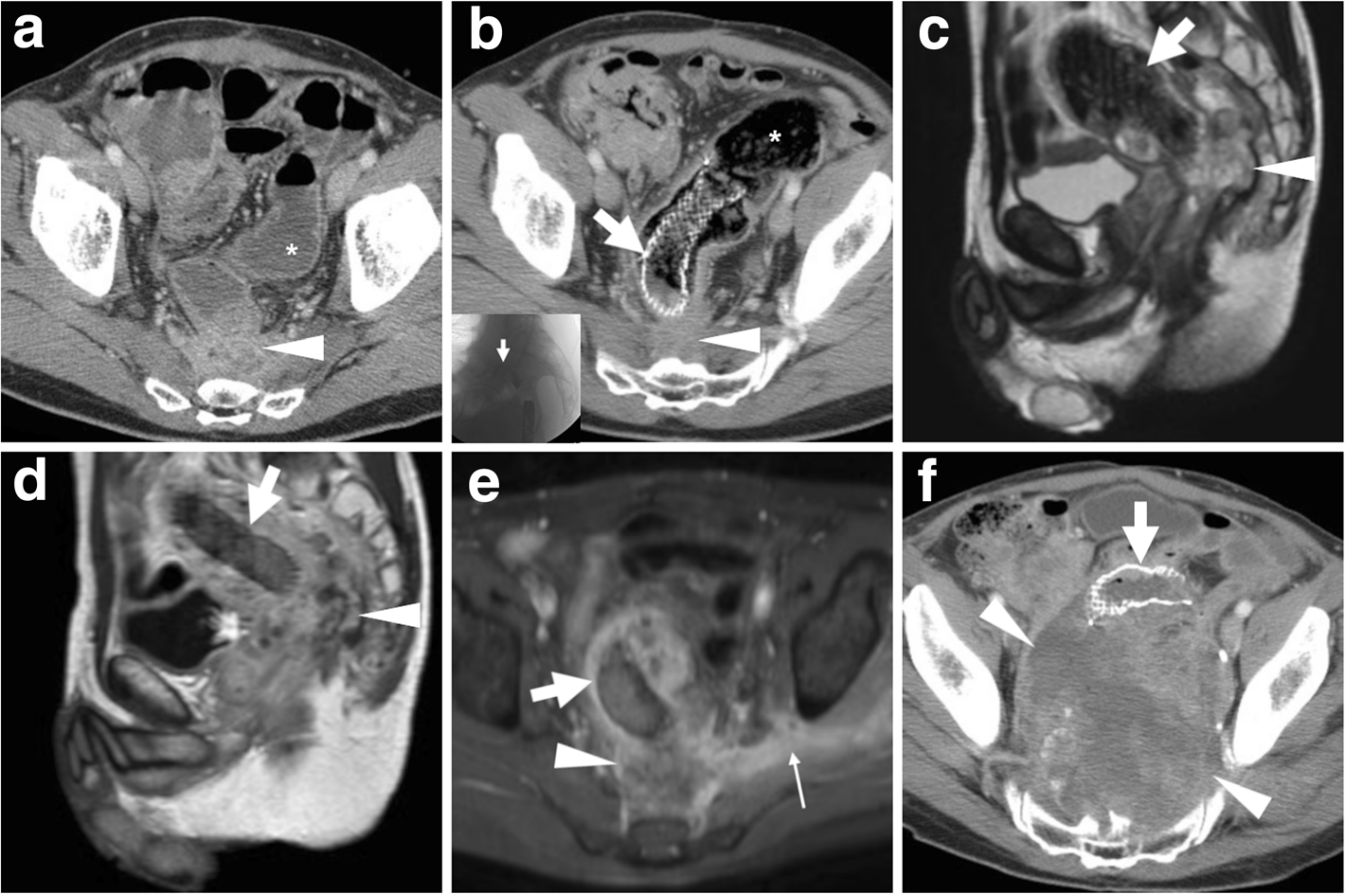
Stent management of an extracolonic pelvic tumour and neoplastic outgrowth. After diagnosis of local recurrence (arrowheads in CT, **a**) of previously resected rectal cancer causing upstream bowel obstruction (*), palliative positioning of a SEMS (thick arrows) was performed (post-procedural CT (**b**), intraprocedural fluoroscopy in inset). Despite intensive chemotherapy, follow-up MRI including sagittal T2- (**c**), post-gadolinium sagittal (**d**) and fat-suppressed axial (**e**) T1-weighted images confirmed patent SEMS (thick arrows) and progressive inhomogeneity of tumour mass (arrowheads) consistent with abscess formation and development of fistulisation through the right ischiatic foramen (thin arrow in **e**). The neoplastic recurrence (arrowheads in CT, **f**) ultimately grew to massive size causing ventral dislocation and compression of the SEMS (thick arrow)

LBO may also occur secondary to extraluminal compression from non-CRC malignancies, recurrent pelvic tumours or peritoneal carcinomatosis. According to the ESGE, SEMS placement is a valid alternative to surgery for minimally invasive palliation of malignant extracolonic obstruction (Fig. 9) [5].

However, the technical and clinical success rates of stenting for extracolonic malignancies are inferior compared to those of CRC. Failed SEMS placement is frequent (up to 60%) due to multiple or long strictures [2, 20].

## Stent management of benign colonic disorders

**Fig. 10:**
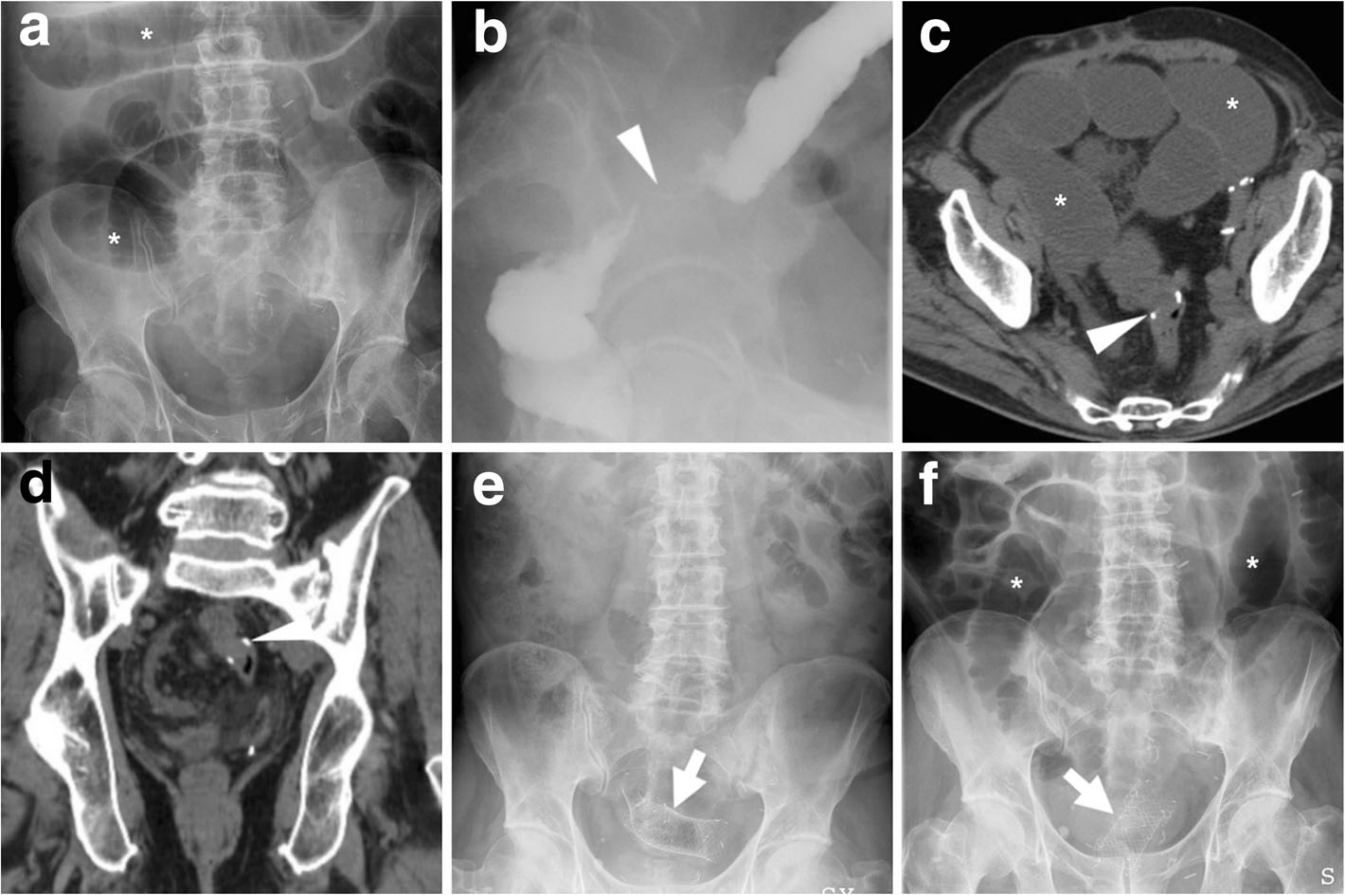
Stent management of a benign anastomotic stricture and SEMS displacement. In a patient with a history of resected sigmoid CRC, plain radiographic (**a**) shows LBO (*). Fluoroscopic contrast enema (**b**) and endoscopy confirm a tight stricture at the site of colorectal anastomosis (arrowhead in **b**) located 12 cm above the anal verge. Noncontrast CT (**c**, **d**) confirms fluid-filled dilated colon (*), without abnormal mural thickening or extraluminal mass at the anastomosis (arrowheads). A SEMS (thick arrows) was placed through the stricture (**e**). Unfortunately, LBO (* in **f**) ultimately recurred after SEMS displacement

Furthermore, colonic stents have been used in a variety of non-malignant conditions including diverticular disease, Crohn’s disease, radiation therapy and anastomotic strictures. On the basis of the limited published evidence, stenting cannot be recommended for most benign colorectal obstructions. Nowadays, the use of SEMS in colonic obstruction from benign causes remains controversial and restricted to selected patients not eligible for definitive surgical treatment, or with strictures refractory or recurrent after endoscopic dilatation (Fig. 10) [21–23].

Some experiences with benign (mostly diverticular and radiation-induced) strictures achieved a high rate of technical success (90%) but limited clinical success (56–87%), due to either acute inflammation or mural stiffness. Additionally, the complication rates (perforation 12–20%, migration 40%, recurrent obstruction 14%) are significantly higher than those observed in the treatment of obstructing CRC [24–26]. Conversely, the use of biodegradable stents or removable covered SEMS is being investigated in patients with anastomotic strictures [6, 7].

## Stent management of post-surgical colorectal complications

Despite advances in modern colorectal surgery, postoperative anastomotic complications (leakage, dehiscence, fistulisation) represent a significant cause of prolonged hospitalisation, morbidity and mortality. The risk of a colonic anastomotic leak (AL) is highest in coloanal and low colorectal anastomoses. Surgical reintervention for complications is associated with additional morbidity and frequent need for faecal diversion: the classic treatment for colorectal or coloanal AL involves removal of the failed anastomosis and creation of a proximal end colostomy (Hartmann’s procedure). Several alternative approaches have been investigated, including endoluminal injection of fibrin sealant, endoscopic clips and transanal closure [27, 28].

**Fig. 11:**
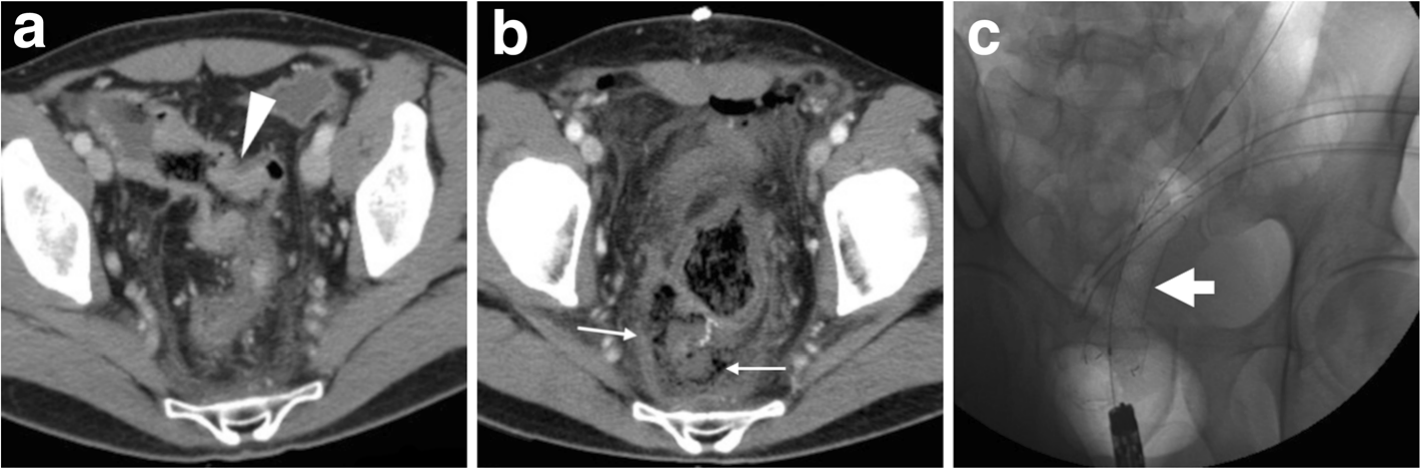
Successful stent management of anastomotic leak (AL). After the CT diagnosis of CRC at the distal sigmoid colon (arrowhead in **a**), an elderly male underwent surgical resection with primary anastomosis. Early postoperative CT (**b**) showed inhomogeneous collection (thin arrows) containing gas bubbles, located between the anterior aspect of the sacrum and the colorectal anastomosis site, consistent with clinical suspicion of AL. The patient ultimately recovered after having a SEMS (thick arrow in **c**) placed through the anastomosis for 3 months

**Fig. 12:**
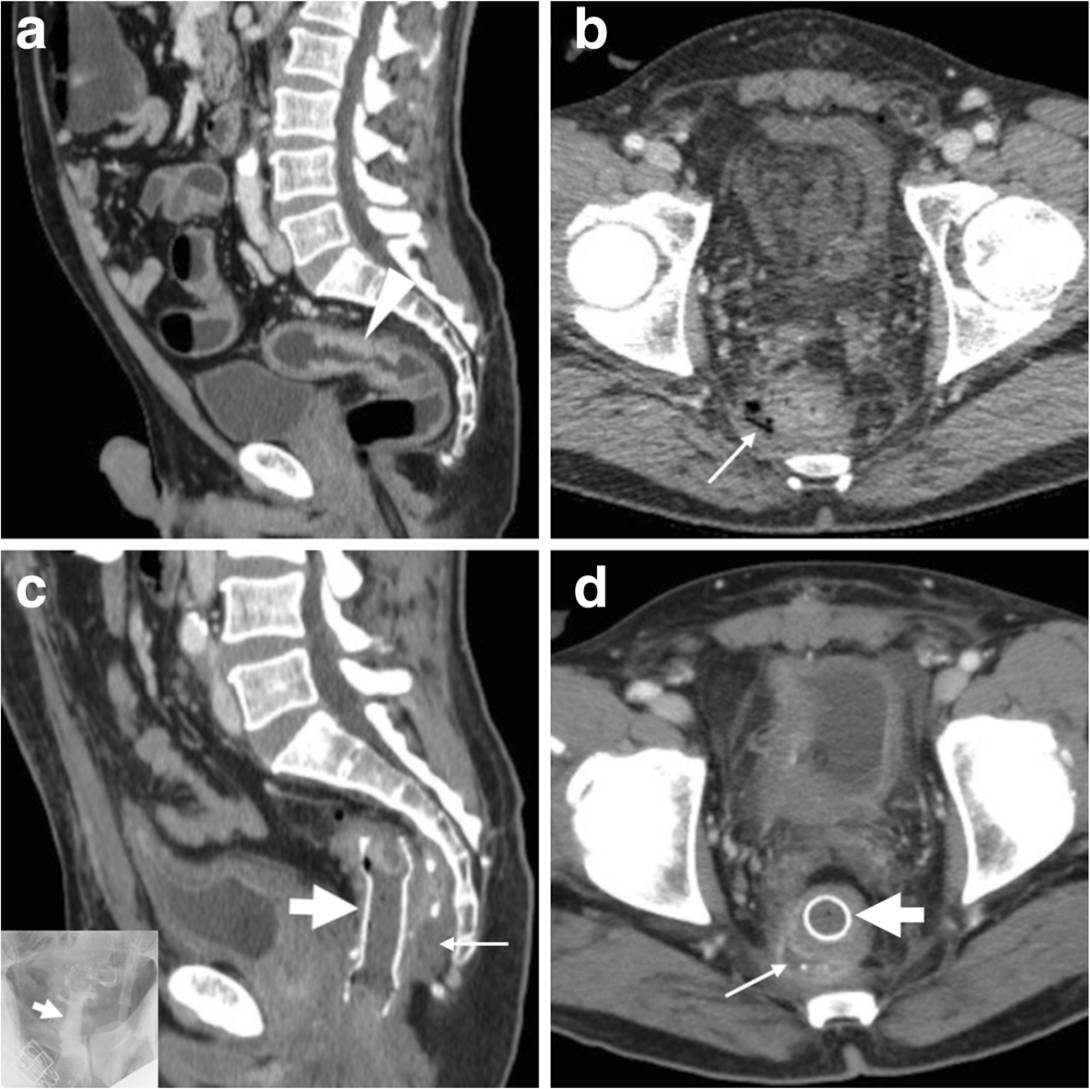
Successful stent management of postoperative AL. Preoperative water enema CT (**a**) showed stricturing CRC of the rectosigmoid junction (arrowhead). Days after anterior rectal resection with an ileostomy, CT (**b**) was requested because of fever and faecaloid material from drainage tube. Gas bubbles (thin arrow) and mild effusion abutting the site of coloanal anastomosis were consistent with the clinical and endoscopic diagnosis of AL, which was treated by placing a covered SEMS through the anastomosis (inset image in **c**). A week later, follow-up CT (**c**, **d**) showed SEMS (thick arrow) in place, minimal residual perianastomotic fluid collection (thin arrows) and the patient ultimately did well.

**Fig. 13:**
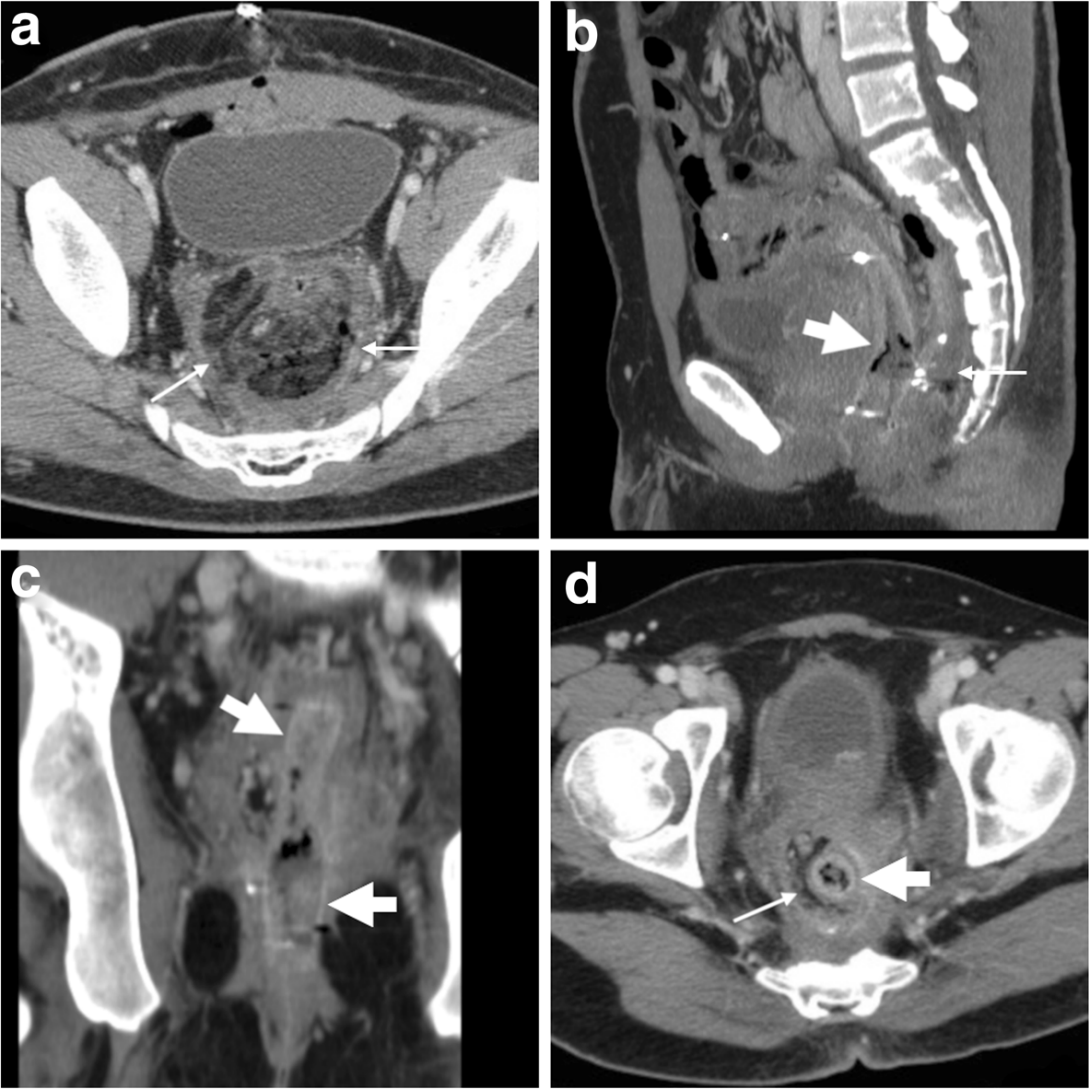
Use of bioabsorbable stent to manage a chronic anastomotic fistula following anterior rectal resection. After drainage of a perianastomotic abscess (not shown), CT (**a**) showed the persistence of perianastomotic inflammation (thin arrows) with gas bubbles (note the close similarity to Fig. 11b). Repeated CT (**b**–**d**) showed decreased perianastomotic inflammation (thin arrows) compared to **a** and confirmed correct placement of the bioabsorbable stent (thick arrows) which appears as a tubular structure with soft-tissue attenuation, best visible using MIP reconstructions (**b**).

The use of endoscopic stenting is increasing in the management of colorectal AL, in patients both with and without a stoma and in combination with percutaneous drainage of abscesses. Covered SEMS (Figs. 11 and 12) or biodegradable stents (Fig. 13) are left in place for 2 months, and the former may be removed once the anastomosis heals. The technical success of stent placement approaches 100%, with a satisfactory clinical success 80–100%. This technique is not an option for very low anastomoses, since the distal end of the stent should be at least 5 cm above the anal verge to limit the risk of migration [23–26, 29]. Additionally, interesting results have been reported in the management of postoperative rectovaginal fistula after CRC resection [30].

## Imaging of colonic stents

### Pre-procedural imaging

**Fig. 14:**
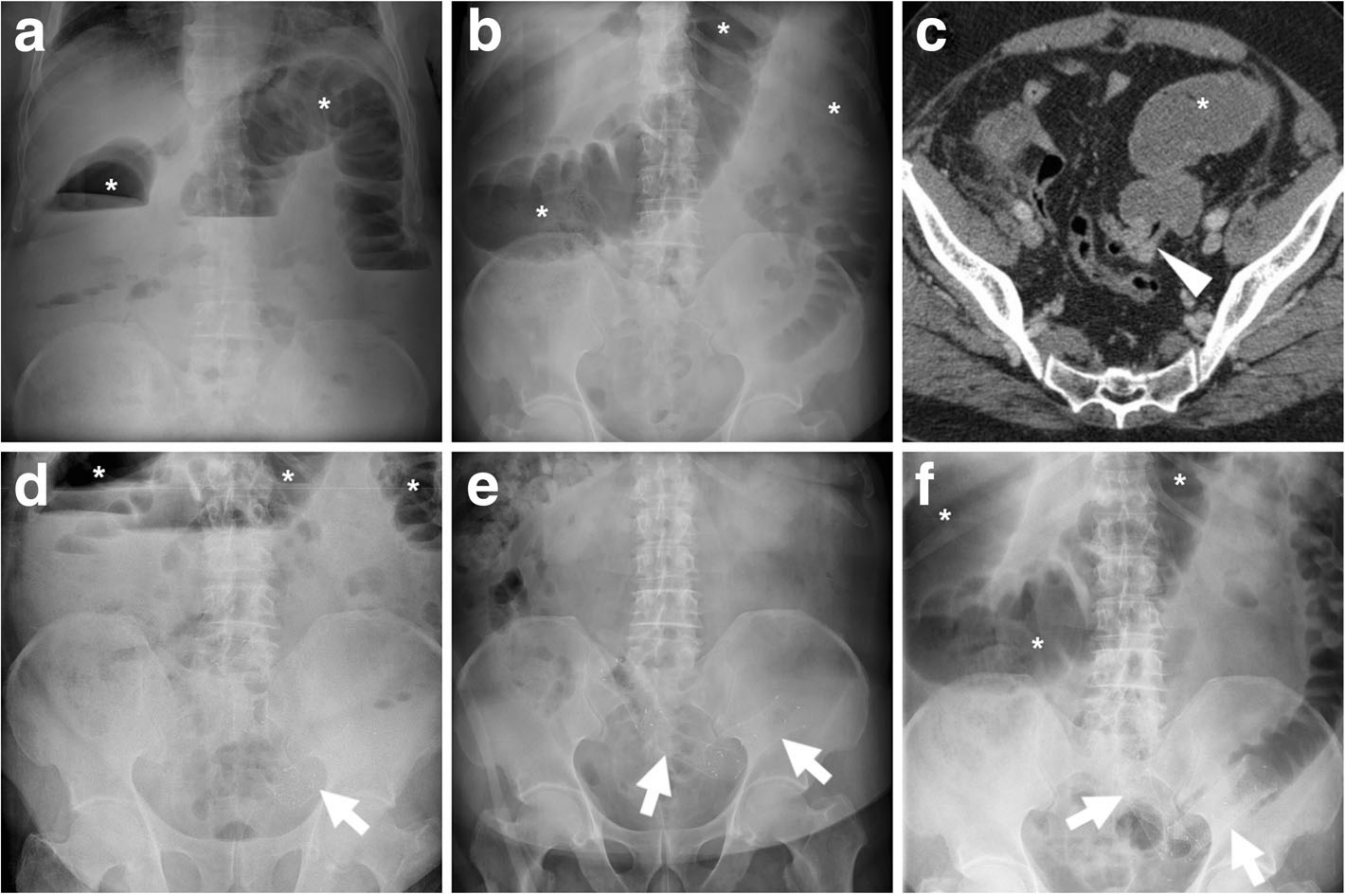
Stent malpositioning causing failed relief of LBO. Initial upright (**a**) and supine (**b**) plain radiographs showed dilated colon (*) with air-fluid levels (**a**) consistent with distal mechanical obstruction. CT (**c**) and endoscopy confirmed a short “apple-core” stricture consistent with sigmoid CRC (arrowhead in **c**). A week after the procedure, radiographic follow-up (**d**-**f**) showed persistently dilated bowel above two angulated SEMS (thick arrows). Immediate surgery was required to remove pT3N0 CRC

**Fig. 15:**
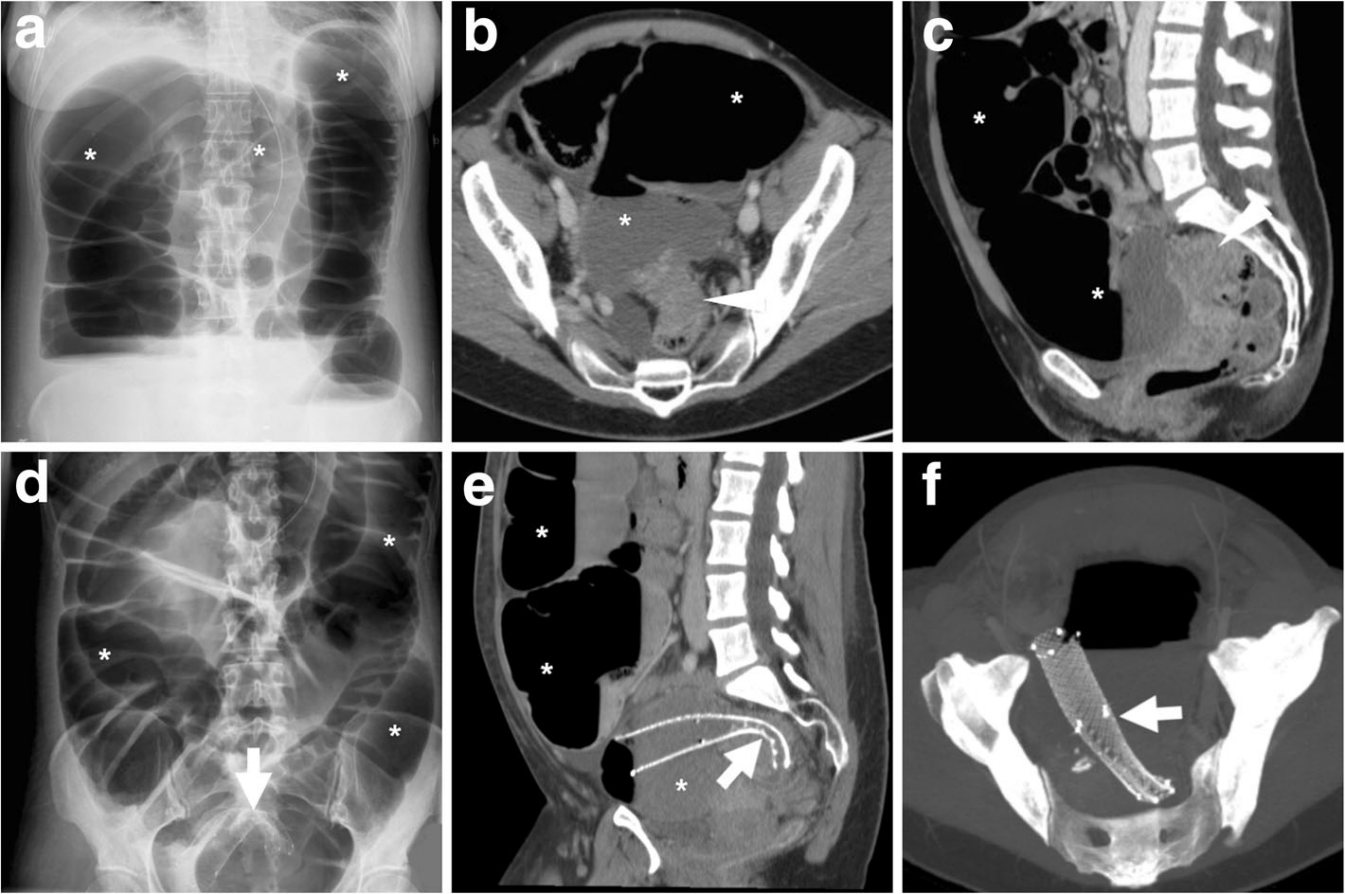
Failed relief of LBO from SEMS treatment of deep infiltrating endometriosis. Initial upright radiograph (**a**) showed severe colonic dilatation (*) with prominent air-fluid levels consistent with high-grade obstruction. Axial (**b**) and sagittal (**c**) CT images confirmed LBO (*) above a segmental mural thickening (arrowheads) at the rectosigmoid junction, which corresponded at pathology to endometriosis. After SEMS (thick arrows) positioning at an impassable extrinsic stricture 15 cm above the anal verge, radiographic follow-up (**d**) and CT (**e**, **f**) showed persistent LBO (*). Sagittal (**e**) and MIP (**f**) CT images showed incomplete expansion of the SEMS (thick arrows). Immediate laparotomy with rectosigmoid resection and temporary stoma was required

Following a clinical and radiographic diagnosis of LBO, the ESGE recognises that contrast-enhanced CT is the mainstay, almost mandatory diagnostic modality for further investigation [5]. Using multiplanar image review, CT is crucial to confirm the presence, site (transition zone), degree of upstream dilatation and underlying cause of LBO (Figs. 3a, 6, 7, 8, 9, 10, 14 and 15). Specifically, the attending radiologist should focus on the detection of obstructing CRC, which appears as either circumferential non-stratified enhancing mural thickening or eccentric, more or less mass-forming solid tissue.

To allow a correct choice and planning of SEMS placement, the endoscopist should be provided with a measurement of length and mural thickness of the colonic stricture plus a semiquantitative assessment of upstream bowel dilatation (including maximum diameter). Additional imaging findings that should be reported as they may contraindicate SEMS placement include multiple sites of obstruction, free perforation and peritonitis, rectal carcinoma and right-sided LBO [4, 31].

### Post-procedural radiographs

Plain radiographs of the abdomen and pelvis are routinely obtained to confirm correct SEMS deployment and to monitor relief of LBO (Figs. 7c and 8b). When interpreting early post-procedural radiographs, radiologists should compare the position of the stent with the previous site of obstruction as depicted in initial endoscopy and CT.

Serial radiographs are often required because SEMS may not fully expand within 24–48 h. The normally patent SEMS becomes nearly cylindrical in shape. When placed at a high-grade stricture, a sufficiently patent SEMS may often remain somewhat “compressed” at its centre. Additionally, in order to confirm effective relief of LBO by stent placement, radiologists should compare the degree of upstream bowel dilatation, number and length of air-fluid levels with initial studies. In high-grade obstruction, the resolution is generally slower but progressive over a few days (Fig. 8).

Later on, plain radiographs become helpful for surveillance of SEMS position, particularly to confirm or exclude stent migration and recurrent LBO [3, 4].

### CT techniques and role

The use of CT provides a comprehensive high-resolution visualisation of the stent, the treated colonic disease and surrounding structures. Knowledge of the stent type, features and indication for placement is helpful for correct interpretation. Reconstruction of focused oblique CT images along the craniocaudal and lateral orientation of the SEMS (Fig. 3) effectively depict its position and course along the large bowel, features and patency. The typical reticular wall (“mesh”) hyperattenuating structure of metal and alloy SEMS is readily identified, best using ample (bone or lung) window settings (Fig. 3d, e). Performing maximum-intensity projection (MIP, Fig. 3e) or three-dimensional volume-rendering images is particularly helpful to analyse the stent’s shape and integrity. Compared to the usual SEMS, biodegradable stents are non-radiopaque and faintly hyperattenuating on CT images (Fig. 13) and therefore best visualised using focused slab MIP images. In normal conditions, patent SEMS show bowel gas and some fluid in the lumen. The CT report should include the presence of intraluminal blood, faeces or solid tissue, the thickness of the diseased colonic segment or the presence of an extraluminal mass and possible signs of complications such as free intraperitoneal air, retroperitoneal gas or fluid/abscess collections [3, 4].

After obtaining effective relief of LBO with a SEMS, comprehensive local, regional and distant staging of CRC is warranted before elective surgery. At this time, either air/CO_2_ CT-colonography or water enema CT (Fig. 5) is useful for preoperative assessment of the colon proximal to the structuring CRC, to detect the possible presence of synchronous tumours or polyps [32–34].

### MRI of colonic stents

In recent years, radiologists are beginning to use MRI to investigate patients with colonic stents in place: SEMS such as the WallFlex™ (Boston Medical) are MRI conditional, but the safety information provided by the manufacturer allow safe imaging under the usual conditions provided by pelvic acquisition protocols on commercial MRI scanners with static magnetic field up to 3 Tesla, with minimal temperature rise and acceptable specific absorption rate (SAR). Multiplanar T2-weighted images and post-gadolinium T1-weighted MRI images effectively depict the treated colonic disease and surrounding structures (Fig. 4). Whereas metal SEMS are intrinsically non-visualised, the patent lumen is devoid of signal, thus allowing to detect the presence of solid, enhancing tumour within the stent, and to correctly interpret abnormal pericolonic tissue as either CRC or abscess (Fig. 9).

## Imaging of stent-related complications

Colonic stenting is generally considered a low-risk procedure, with a 20% overall morbidity rate, mortality below 1% and occasional need for surgery and stoma. Listed in Table 2, complications associated with colonic SEMS can be categorised as either early or late and may develop in both palliative and bridge-to-surgery settings. Risk factors for post-procedural complications include small SEMS caliber (≤ 22 mm), complete LBO, proximally located (right colon) CRC, extracolonic lesions, concurrent stricture dilation, history of radiation and chemotherapy [10, 12, 15, 35].

**Table 2:** Complications after stent placement in the management of colorectal disorders

Early complications	Late complications
Technical failure	Stent migration
Stent misplacement/failed relief of obstruction	Re-obstruction
Haemorrhage	(Chemotherapy-related) perforation
Stent migration	Fistulisation
Perforation	

Patients with colonic SEMS commonly complain of symptoms such as pelvic or rectal pain. In the majority of cases, CT is the technique of choice to comprehensively investigate patients with clinical suspicion of stent-related complications [3, 4].

### Failed relief of LBO

Shortly after stent placement, the lack of clinical improvement and persistent imaging evidence of colonic dilatation and air-fluid levels may result from either malpositioning or incomplete expansion of the SEMS. The imaging diagnosis of misplacement (Fig. 14) should rely on the discrepancy between the position of the SEMS and the site and length of the treated tumour as shown in previous cross-sectional studies. Sometimes, the SEMS is positioned too distally and does not cover the entire length of the structure. On the other hand, failed expansion (Fig. 15) is easily assessed on both radiographs and CT, when the SEMS remains “hourglass-shaped.” In situations, endoscopic reintervention or emergency surgery is required to manage obstruction [3, 4].

### Haemorrhage

**Fig. 16:**
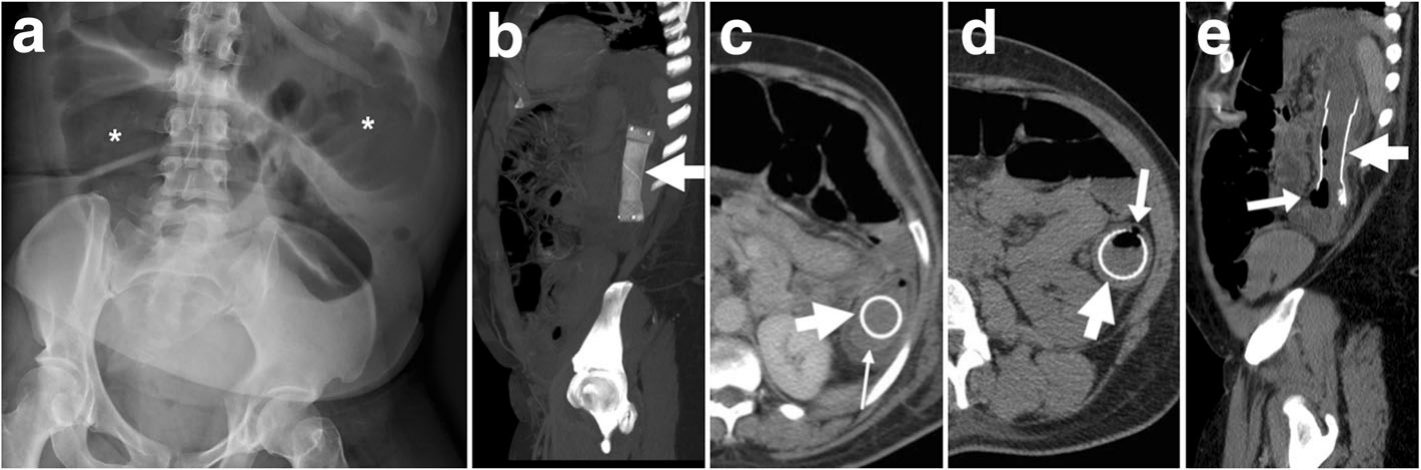
Haemorrhage and impending stent-related perforation. Initial radiograph (**a**) showing marked colonic dilatation (*). After early clinical improvement, the patient experienced haematochezia. MIP CT image (**b**) confirmed correctly placed and expanded SEMS (thick arrows) at the descending colon. CT images showed some hyperattenuating fresh blood (thin arrow in **c**) in the bowel lumen and a gas bubble (arrows in **d** and **e**) piercing through the colonic wall at the distal end of the SEMS. Emergency laparotomic surgery (left hemicolectomy with primary anastomosis) was required

Self-limited bleeding occurs in 5% of cases and is generally minor, without the need for specific treatment. The CT hallmark is the identification of hyperattenuating material consistent with fresh blood in the bowel lumen (Fig. 16) on precontrast scans [3, 4].

### Perforation

Although uncommon, perforation is the most feared SEMS-related complication with non-negligible mortality (approximately 10%). Albeit incidence figures are highly variable among different studies and settings, the reported frequency is below 5% of all patients. Specific risk factors for early perforation following SEMS placement include previous irradiation, balloon dilation prior to stent insertion and excessive manipulation of guidewires, especially in the presence of diverticular disease or colonic wall ischaemia [36]. On the other hand, late perforations developing 3 to 6 months after stent insertion are generally associated with systemic (mostly bevacizumab) chemotherapy. Surgery should always be considered in patients with SEMS-related perforation [5].

Often clinically silent, colonic perforation is almost invariably diagnosed by CT. Imaging findings to be reported as consistent with perforation include nondependent pneumoperitoneum, free peritoneal effusion, pericolonic gas bubbles or fluid collections. However, in most cases, the amount of extraintestinal air is very limited (Fig. 16) [3, 4].

### Stent migration

**Fig. 17:**
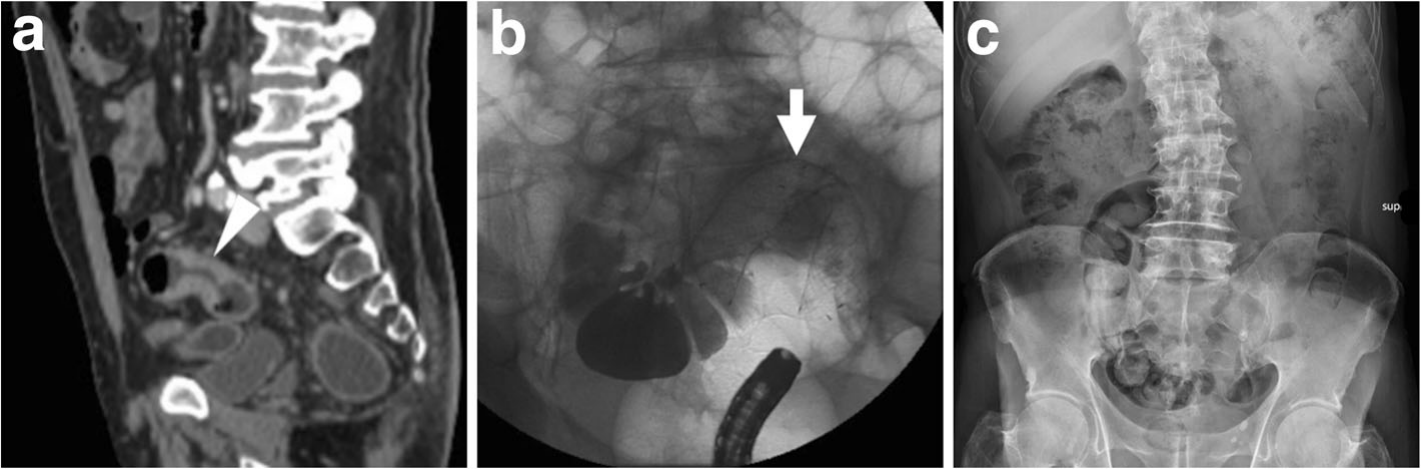
Spontaneous stent migration. In a patient with endoscopically impassable sigmoid CRC (arrowhead in initial water enema CT, **a**) with liver metastases (not shown) at diagnosis, multidisciplinary consultation opted to postpone surgical resection after neoadjuvant chemotherapy. A SEMS (thick arrow) was positioned endoscopically under radiologic guidance (**b**) and allowed relief of LBO, clinical improvement and start of chemotherapy. A month later, the patient experienced evacuation of the SEMS with stools and radiographs (**c**) confirmed SEMS loss [adapted from Open Access Ref. [38]

Migration represents the commonest complication (overall incidence 10–11%, up to 50% of patients in some series) and may occur at any time following the SEMS insertion. Migration tends to develop with stents which are too small in diameter and/or too short compared to the stricture, is much more likely with fully covered compared with uncovered SEMS and can be favoured by chemotherapy-induced tumour shrinkage. Other factors that predispose to stent migration include treatment of partial LBO, extracolonic masses and tumours of the distal rectum. It may either remain asymptomatic or lead to further complications such as recurrent obstruction or perforation. Most usually, displaced stents generally migrate distally and are expelled through the anus. In these cases, repeated radiographs fail to detect the presence of the SEMS (Fig. 17). Alternatively, stent displacement (Fig. 10e, f) is judged on the basis of accurate comparison of the SEMS position and orientation compared to previous studies: without a baseline image, the diagnosis may be missed. Symptomatic occurrences may require endoscopic repositioning, removal or replacement [2, 10, 12, 35, 37].

### Stent re-obstruction

**Fig. 18:**
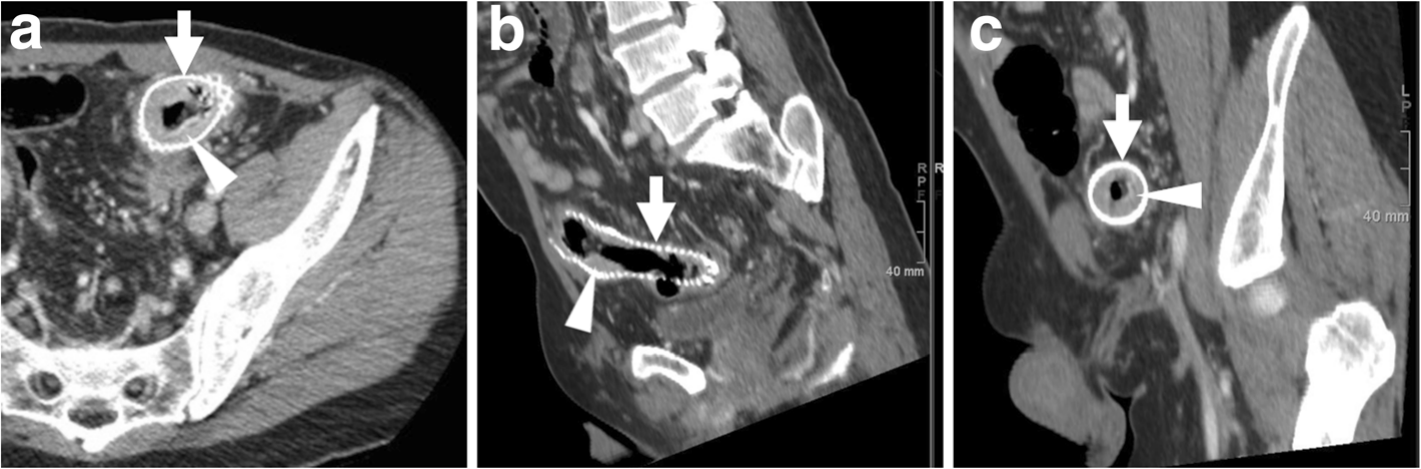
Tumour ingrowth causing stent malfunctioning. In the same patient, compared to initial MRI (Fig. 5), focused multiplanar reconstructions (**a**-**c**) of follow-up CT showed enhancing neoplastic tissue (arrowheads) growing within the SEMS (thick arrows), leading to recurrent LBO

The use of CT is crucial to differentiate between possible causes of recurrent LBO. Mostly observed in the palliative setting, re-obstruction is heralded at imaging by an increased amount of air-fluid bowel dilatation above the SEMS. In descending order of frequency, causes include tumour ingrowth or outgrowth, stent migration (Fig. 10), luminal impaction with stools and occasional SEMS fracture or angulation. Ingrowth (i.e. neoplastic tissue crossing through the “mesh” of an uncovered SEMS, Fig. 18) is diagnosed when CT or MRI detects the presence of solid enhancing tumour within the stent lumen and may be managed using laser/argon therapy, additional “stent-in-stent” placement or surgery. Conversely, outgrowth (Fig. 9) refers to the progression of neoplastic tissue surrounding or at the extremities of the stent. Faecal occlusion can be prevented using a dedicated low-residue diet and osmotic laxatives, appears at CT as SEMS lumen is filled with more or less thick faecal material with intermixed gas bubbles (Fig. 3b, c) and may require medical or endoscopic desobstruction. Mechanical malfunction requires removal or replacement of the SEMS [2–4].

## Conclusion

Nowadays, SEMS represent an established alternative to surgery for palliation of unresectable CRC and are widely used as a bridge-to-surgery to relieve LBO before elective oncologic resection. Additionally, intraluminal stents represent an appealing therapeutic option to manage selected benign colorectal disorders, particularly post-surgical strictures and leaks. At hospitals performing advanced multidisciplinary, radiologists should be aware of the technical basics and usual imaging appearances of colorectal stenting, in order to provide appropriate pre- and post-procedural studies and detect possible stent-related complications.

## Abbreviations

AL: Anastomotic leak
ASA: American Society of Anesthesiologists
CRC: Colorectal cancer
CT: Computed tomography
LBO: Large bowel obstruction
MIP: Maximum-intensity projection
MRI: Magnetic resonance imaging
SEMS: Self-expanding metal stent
